# Astragalin Attenuates Bone Destruction and the Progression of Bone Metastasis in Breast Cancer

**DOI:** 10.3390/cancers17213442

**Published:** 2025-10-27

**Authors:** Sizhen Yang, Ying Zhang, Hao Qiu, Xu Hu, Tongwei Chu

**Affiliations:** Department of Orthopedics, Xinqiao Hospital, Army Medical University, Chongqing 400037, China; ysz2016@tmmu.edu.cn (S.Y.); zyaspine@tmmu.edu.cn (Y.Z.); qiutmmu@tmmu.edu.cn (H.Q.)

**Keywords:** astragalin, breast cancer, osteoclastogenesis, bone metastasis

## Abstract

**Background:** Bone metastasis of breast cancer (BC) is a key reason for poor prognosis. Recently, natural ingredients derived from plants have been found to exert a broad anti-tumor effect and are considered to be promising candidates for adjuvant therapy. Astragalin (AS) was found to inhibit the progression of several types of tumors; however, the role of AS in regulating the bone metastasis of BC is still unclear. **Methods:** The effects of AS on the progression of bone metastasis of BC were detected in vivo through safranin O and fast green staining, in vivo living imaging and microCT. The BrdU assay and Annexin V-PI analysis were used to detect the effects of AS on the growth of BC cells. Furthermore, TRAP staining was performed to examine the formation of osteoclasts regulated by AS. A transcriptome was performed to explore the downstream effects of AS on regulating the growth of BC cells, and the mechanism was further confirmed by Western blot and real-time PCR. **Results:** Administration of AS could effectively attenuate the bone destruction and the progression of bone metastasis of BC. The growth of BC cells can be inhibited by AS by inducing ER stress-mediated upregulation of Ddit3. In addition, AS can also prevent osteoclastogenesis through inhibiting the activation of the AKT pathway. **Conclusions:** Our studies suggest that AS could be an ideal adjuvant therapy for attenuating the progression of bone metastasis of BC, since it can directly restrict the growth of tumor, as well as attenuate osteolysis.

## Simple Summary

Astragalin (AS) showed potential anti-tumor properties in various cancers; however, it is still unclear whether it affects the progression of bone metastasis in breast cancer (BC). In this study, AS markedly attenuated bone destruction and tumor growth. Further studies confirmed that AS can regulate the development of BC cells and osteoclasts. Flow cytometry indicated that AS can induce the apoptosis of both MDA-MB-231 and 4T1 cells in vitro. Transcriptome data indicated that Ddit3 was significantly upregulated by AS. Consistently, Ddti3 knockdown reversed the effects of AS on BC cells. Mechanically, AS-induced ER stress inhibited the activation of the AKT pathway. On the other hand, AS also directly inhibited osteoclastogenesis partly by inhibiting AKT. Our study revealed that AS is a promising treatment for bone metastasis in BC.

## 1. Introduction

Bone is one of the most common metastatic sites of breast cancer (BC) [1,2]. Patients with BC often suffer from a worse prognosis after bone metastasis. Bone metastasis of BC presents with osteolytic lesions, leading to hypercalcemia, pathological fracture, pain, and worse quality of life and prognosis. Finding a safe and effective therapy is critical to attenuate the progression of bone metastasis of BC and to prolong the lifetimes of patients.

The aberrant activation of osteoclasts (OCs) and their precursors (OCPs) is the key reason for osteolysis and the progression of bone metastasis of BC [3]. In the tumor microenvironment, OCPs differentiate into OCs in a RANK/RANKL-dependent or -independent manner [4,5]. Various cell types, such as tumor cells and stromal cells, modulate the formation and activation of OCs by releasing cytokines [5,6,7,8]. Several dominant pathways, including NF-κB, PI3K/AKT, JAK/STAT, and MAPK pathways, regulate the differentiation and function of OCs [5,9,10,11]. Consistently, for bone metastasis of BC, the dominant pharmacotherapies in clinics focus on targeting osteoclastogenesis. The two most commonly used therapies for bone metastasis of BC, denosumab and zoledronate, both exert their effects to inhibit osteolysis by targeting OCs. In theory, therapies targeting both the tumor microenvironment and tumor cells are ideal for improving the therapeutic effects on varieties of cancers. However, current therapeutic methods targeting cancer cells that colonize bone tissue are limited in the treatment of BC and should be further improved.

As an adjuvant therapy, bioactive ingredients derived from various plants are ideal candidates for treating cancer, improving the curative effects of traditional therapies, and can target both the tumor microenvironment and tumor cells [12,13,14]. Of these, Astragalin (AS), dominantly extracted from Astragalus, is a food-derived flavonoid that exerts therapeutic effects on multiple diseases, including cancer. In Chinese traditional medicine, Astragalus was a common Chinese herb to be used for anti-inflammation, regulating blood glucose and so on. It is stable at room temperature and is easily dissolved in DMSO or methyl alcohol. Previous studies showed AS can attenuate the proliferation of colon cancer cells, kidney cancer cells, lung cancer cells, etc. [15,16,17]. Furthermore, computational/experimental studies have shown that AS can suppress metastasis and angiogenesis in BC [18]. These results imply that AS could be a candidate for directly targeting BC cells while remodeling the tumor microenvironment in bone metastasis of BC. However, the detailed mechanisms of AS inhibiting BC should be further clarified. Therefore, in this study, we investigated the effects of AS on the fate of BC cells, as well as osteoclastogenesis, to verify its effects on the progression of bone metastasis in BC and explore the underlying mechanisms.

## 2. Methods and Materials

### 2.1. Cells and Treatment

Luciferase-labeled MDA-MB-231 and 4T1 cells were established following the manufacturer’s protocol through infection with UBI-MCS-EGFP-SV40-FIREFLY-Luciferase-IRES-Puromycin, and the cells were cultured with 5 μg/mL polybrene. After overnight incubation, the culturing medium was replaced, and the cells were screened in 4 μg/mL puromycin. For transfection with siRNA, BC cells were transfected with siRNA against human or mouse Ddit3 and IRE using INVI DNA RNA Transfection Reagent (Invigentech, CA, USA) for 24 h, as suggested by the manufacturer’s protocol. In some experiments, 20 μM AS was added after changing the culture medium. 

### 2.2. In Vitro Osteoclastogenesis Assays

Cells derived from bone marrow were rinsed out, filtered, and cultured with M-CSF (50 ng/mL) for 24 h. Then, non-adherent cells were transferred to new culture plates and cultured with M-CSF (50 ng/mL) and RANKL (100 ng/mL) to obtain bone marrow macrophages/monocytes (BMMs) [19]. In some experiments, SC79 was added to treat BMMs cells at 20 μM for 24 h, and then AS (20 μM, MedChemExpress LLC) or its vehicle, DMSO, was added. When detecting osteoclast formation, the cells were fixed in 4% paraformaldehyde for 30 min and then stained with TRAP staining solution (Wako, Japan), following the manufacturer’s instructions.

### 2.3. Quantitative Real-Time PCR 

Total RNA was extracted using TRIzol Reagent (Invitrogen, Carlsbad, CA, USA). RNA was reverse transcribed using PrimeScript™ RT Master Mix (Takara Bio, Shiga, Japan), following the manufacturer’s instructions. cDNAs were used for detecting mRNA levels using the TB Green Premix Ex Taq II (Takara Bio, Shiga, Japan). GAPDH was used as the internal control. Primer sequences were as follows: human GAPDH (forward: 5′-AGACCTGTACGCCAACACAG and reverse: 5′-TTCTGCATCCTGTCGGCAAT-3′), mouse GAPDH (forward: 5′-TGTGTCCGTCGTGGATCTGA-3′ and reverse: 5′-CCTGCTTCAC CACCTTCTTGAT-3′), human IRE1 (forward: 5′-AATTGTGTACCGGGGCATGTc and reverse: 5′-TTCTCCGTGCAGAAGTAGCG-3′), mouse IRE1 (forward: 5′-CTGGCTTC TCATAGGACACCAT-3′ and reverse: 5′-TCTCGATGTTTGGGCAGGTT-3′), human Ddit3 (forward: 5′-ACCTCCT GGAAATGAAGAGGAAG-3′ and reverse: 5′-CAGTCAGCCA AGCCAGAGAA-3′), mouse Ddit3 (forward: 5′-GCTGGAAAGCAGC GCATGAA-3′ and reverse: 5′-GCGAGTCGCCTCTACTTCCC-3′), human ki67 (forward: 5′-TCCTTTGGTGGGCACCTAAGACCTG-3′ and reverse: 5′-TGATGGTTGAGGTCGTTCCTTG ATG-3′), mouse ki67 (forward: 5′-ACCATCATTGACCGC TCCTT-3′ and reverse: 5′-TTGACCTTCCCCATCAGGGT-3′), human Bcl2 (forward: 5′-GGGGTCATG TGTGTGGAGAG-3′ and reverse: 5′-GTTCCACAAAGGCATCCCAG-3′), mouse Bcl2 (forward: 5′-GACTGAGTACCTGAACCGGC-3′ and reverse: 5′-AGTTCCACAAAGGCAT CCCAG-3′), mouse PU.1 (forward: 5′-CCCGGAGTTCGACTTCGATT-3′ and reverse: 5′-TAACTGTAGTGTTCTGCGGC-3′), and mouse Oc-stamp (forward: 5′-ACTCA CAGTCAAATATG ACGCCT-3′ and reverse: 5′-GTAGAT GACAGTC GTGGGGC-3′).

### 2.4. Application of Small Interfering RNA (siRNA)

Small interference RNAs (siRNAs) targeting Ddit3(si-Ddit3) and IREI (si-IREI) were synthesized by GenePharma Co., Ltd. (Shanghai, China). The target sequences were as follows: negative control sense: 5′-UUCUCCGAACGUGUCACGUTT-3′ and antisense: 5′-ACGUGACACGUUCGGAGAATT-3′; si-Ddit3 sense: 5′-GGCAGCCACUUCAGCGAUCGA-3′ and antisense: 5′-UUUAUGUGA AGUGGAUCGCTT-3′; and si-IREI sense: 5′-ACUGGAGUUGUACGGCACGUG-3′ and antisense: 5′-UGUUGAGGCAGAGAAGGCUUG-3′. The cells were transfected with 100 nM siRNAs using Lipofectamine 2000 (Invitrogen, Carlsbad, CA, USA), following the manufacturer’s instructions. After transfection for 48 h, the mRNA and protein levels were analyzed.

### 2.5. Western Blotting

Total protein was subjected to SDS-PAGE and transferred to a polyvinylidene difluoride (PVDF) membrane. Then, the membrane was blocked with 5% BSA and incubated with antibodies against Ddit3 (Affinity, AF6277,1:1000, Cincinnati, OH, USA), phosphorylated IRE1 (Affinity, AF7150,1:1000, Cincinnati, OH, USA), total protein of IRE1 (Affinity, DF7709,1:1000, Cincinnati, OH, USA), phosphorylated AKT (Affinity, AF0016, 1:1000, Cincinnati, OH, USA), total protein of IRE1 (Affinity, AF0836, 1:1000, Cincinnati, OH, USA), phosphorylated p38 MAPK (Affinity, AF4001, 1:1000, Cincinnati, OH, USA), total protein of IRE1 (Affinity, AF6456, 1:1000, Cincinnati, OH, USA), and β-actin (Affinity, AF7018, 1:1000, Cincinnati, OH, USA). Next, the samples were incubated with HRP-conjugated IgGs (ZSGB-Bio, ZB-2301, 1:5000, Beijing, China). Subsequently, the membranes were incubated with HRP-conjugated IgGs. The target proteins were assessed using an ECL system and were visualized using the ChemiDoc system (Bio-Rad, Hercules, CA, USA).

### 2.6. In Vitro Proliferation Assay and Apoptosis Assay

For the proliferation assay, the BrdU solution was added to the culture medium and incubated for 2 h. The rate of BrdU incorporation was detected using the Phase-Flow BrdU kit (Biolegend, San Diego, CA, USA), following the manufacturer’s instructions. For the apoptosis assay, the cells were detected using Annexin V/PI kit (BestBio, Shanghai, China), based on the manufacturer’s instructions. The BrdU assay and apoptosis assay were conducted via flow cytometry and using Beckman Cytoflex (Indianapolis, IN, USA).

### 2.7. Animal Experiments

Eight–ten-week-old female mice were used in this study. The mice were anesthetized with an intraperitoneal injection of 0.5% (*w*/*v*) pentobarbital sodium to induce general anesthesia. To establish the bone metastasis model, 5 × 10^5^ tumor cells were resuspended in PBS and injected into the tibias of anesthetized female wild-type or BALB/c-nu/nu nude mice. AS was intraperitoneally (i.p.) injected for 5 consecutive days at a concentration of 20 mg/kg, the same volume of DMSO was used as a control. Bioluminescence images were captured 3 weeks after injecting BC cells. Histochemical analysis and μCT analysis were conducted 3 weeks after injecting BC cells. For the in vivo BrdU assay, cancer cells were labeled with Dil (1 µL per 1 mL culture medium) for 1 h. Then, Dil-labeled cells were injected into the tibias (200,000 cells per leg). Furthermore, 200 µL of BrdU solution (10 mg/mL) was intraperitoneally injected 12 h and 3 h before harvesting. Then, BrdU incorporation in cancer cells was detected via flow cytometry using the gating strategy (Dil-PE+: BrdU-FITC-) [20]. For the dose, since BrdU assay was detected at an early stage; thus, AS was injected only once, and we increased the dose. After reaching the detection time point, the mice were euthanized using the carbon dioxide asphyxiation method. Animal experiments were approved by the Laboratory Animal Welfare and Ethics Committee of the Army Medical University (approval no. AMUWEC20245293).

### 2.8. Bioluminescence Imaging (BLI)

Male C57BL/6 and nude mice aged 6 to 8 weeks were used for experiments. To establish the bone metastasis model, BC cells were injected into the tibia following a previously described standard method [21]. Mice were intraperitoneally injected with 75 mg/kg D-Luciferi. Bioluminescence images were acquired by using the IVIS Imaging System (Perkin Elmer, Waltham, MA, USA) after 2–5 min. Analysis was conducted by measuring photon flux within the interest area.

### 2.9. M. CT Analysis

A Bruker Skyscan 1174 X-ray microtomograph system (Kontich, Belgium) with an isotropic voxel size of 12.0 μm was used to capture the images of the tibias. Scans were conducted in 4% paraformaldehyde, with an X-ray tube potential of 50 kV and an intensity of 800 μA. For trabecular bone analysis, a 0.6 mm region, 0.1 mm below the growth plate to a 0.6 mm distance, was contoured. All images represent the respective groups. 

### 2.10. Transcriptome

Total RNA was extracted from 4T1 cells that were treated with or without AS for 24 h. RNA-seq was conducted by Phalanx Biotech Group (Taiwan, Zhubei City, China). The probe content was in accordance with version 19.0 of the Sanger miRBase database. The signal intensity of each spot was calculated using the R program (2.12.1). The median value of repeating spots was selected for future analysis. We filtered out the spots, which flagged < 0 within the array. Spots passing the criteria were normalized using the invariant set normalization method. Normalized spot intensities were transformed into gene expression log2 ratios between the control group and treatment group using the pair-wise *t*-test. The spots with a |log2 ratio| ≥ 0.585 and a *p* value < 0.05 were selected for analysis. Differential gene expression was identified using DESeq2 (v1.36.0) in R (v4.2.0), with significance defined as FDR < 0.05 and |log2FoldChange| > 1. The resulting DEGs were used as inputs for GO, KEGG, and GSEA functional enrichment analyses conducted using clusterProfiler (v4.4.4). The token is cradqwqkjxglhcr.

### 2.11. Histochemical Analysis

Bone tissues were decalcified using tetrasodium EDTA. Tissue specimens were dehydrated by passage through an ethanol series, cleared twice in xylene, embedded in paraffin, and sectioned at 8–10 μm thickness. For safranin O-fast green staining, the sections were immersed in 0.1% safranin O solution for 3 min. Then, the samples were immersed in 0.1% fast green solution for 1 min. Color separation was conducted using 1% acetum. 

### 2.12. Statistical Analysis

Data are presented as the mean ± SD per experimental condition. Differences between two groups were evaluated using a two-tailed Student’s *t* test. More than two groups were compared using one-way ANOVA, followed by a post hoc Dunnett’s test. Differences with *p* < 0.05 were considered statistically significant.

## 3. Results

### 3.1. AS Inhibited the Progression of Bone Metastasis in BC

**Figure 1:**
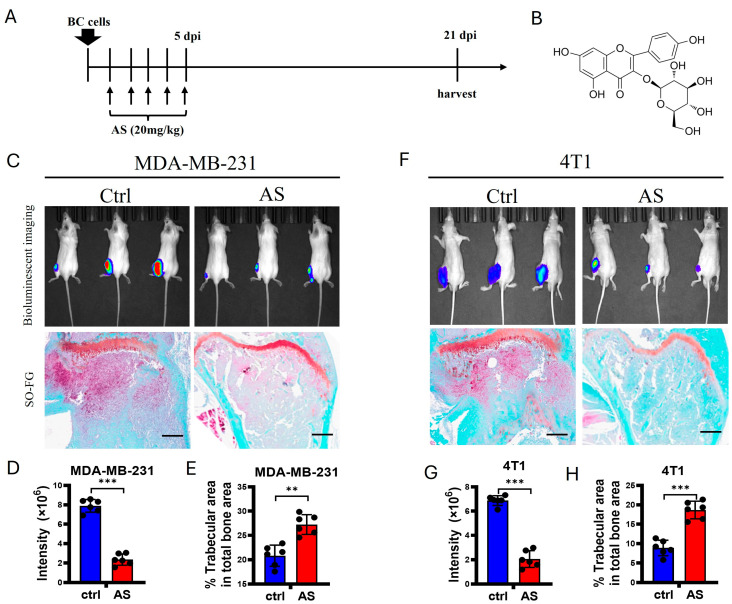
AS attenuated the progression of bone metastasis in BC. (**A**) Schematic diagram showing experimental models of bone metastasis in BC treated with AS. (**B**) The structure of AS. (**C**) Local inoculation of MDA-MB-231 cells into the tibias of nude mice, followed by treatment with AS. Representative bioluminescence images at 3 weeks (**upper**) and safranin O-fast green staining of tibias at 3 weeks (**lower**, scale bar = 100 μm) (*n* = 6 mice per group for BLI; *n* = 6 mice per group for histochemical staining). (**D**,**E**) Quantification of bioluminescence signaling intensity in C (**D**) and the percentage of trabecular area in C (**E**). (**F**) Wild-type (WT) mice locally inoculated with 4T1 cells in their tibias and treated with AS. Representative bioluminescence images (**upper**) and safranin O-fast green staining of tibias at 3 weeks (**lower**, scale bar = 100 μm) (*n* = 6 mice per group for BLI; *n* = 6 mice per group for histochemical staining). (**G**,**H**) Quantification of bioluminescence signaling intensity in F (**G**) and the percentage of trabecular area in F (**H**). Ctrl, control group; BC, breast cancer; AS, astragalin; dpi, days after injection. ** *p* < 0.01; *** *p* < 0.001.

To detect whether AS affects bone metastasis in BC, 4T1 cells, a murine BC cell line, and MDA-MB-231 cells, a human BC cell line, were injected into the tibias of Balb/c mice and nude mice, respectively. Then, AS was injected intraperitoneally for 5 consecutive days (Figure 1A,B). Using bioluminescence imaging (BLI), we found that the lesion sizes at the tibias of mice were significantly decreased after treatment with AS. Consistently, trabecular area was preserved after treatment with AS (Figure 1C–H). Taken together, these findings indicate that AS attenuated bone metastasis in BC. 

### 3.2. AS Inhibited the Growth of BC Cells Both In Vitro and In Vivo

**Figure 2:**
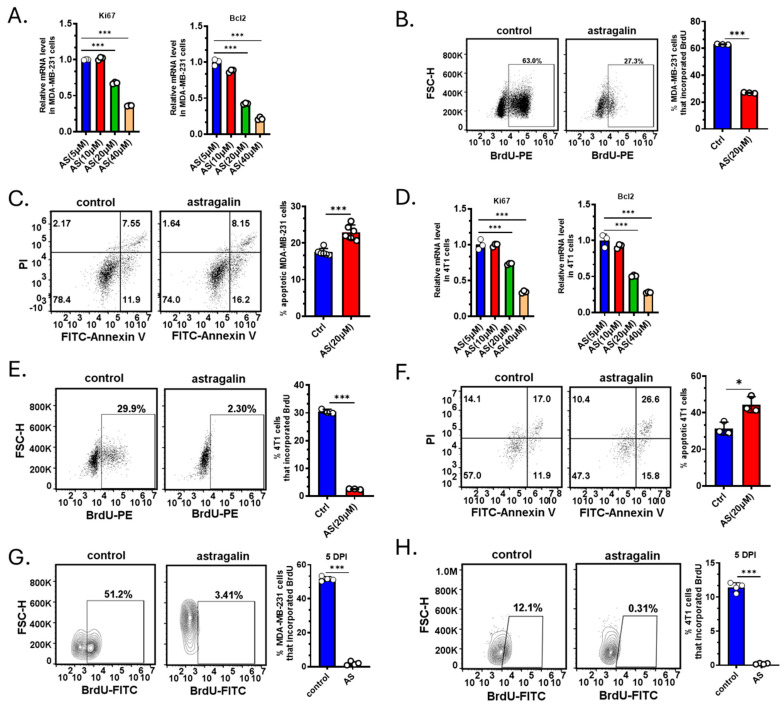
AS inhibited BC cell growth. (**A**) Real-time PCR detecting the mRNA levels of ki-67 and Bcl-2 in MDA-MB-231 cells after treatment with AS (5, 10, 20, and 40 µM) for 48 h. (**B**) Representative flow cytometry images showing BrdU incorporation in MDA-MB-231 cells after treatment with AS (20 µM) or vehicle (**left**) and the quantification results (**right**). (**C**) Representative flow cytometry images showing the apoptotic rate in MDA-MB-231 cells after treatment with AS (20 µM) or vehicle (**left**) and the quantification results (**right**). (**D**) Real-time PCR analysis detecting the mRNA levels of ki-67 and Bcl-2 in 4T1 cells after treatment with AS (5, 10, 20, and 40 µM) for 48 h. (**E**) Representative flow cytometry images showing the proportion of BrdU incorporation in 4T1 cells after treatment with AS (20 µM) or vehicle (**left**) and the quantification results (**right**). (**F**) Representative flow cytometry images showing the apoptosis rate in 4T1 cells after treatment with AS (20 µM) or vehicle (**left**) and the quantification results (**right**). (**G**,**H**) MDA-MB-231 cells or 4T1 cells were labeled with Dil dye and then injected into the tibias. Flow cytometry analysis detecting BrdU incorporation in MDA-MB-231 cells (**G**) or 4T1 cells (**H**) after treatment with AS (40 mg/kg) or vehicle at a specific time point. AS, astragalin; ctrl, control. * *p* < 0.05; *** *p* < 0.001.

Since treatment with AS reduced the tumor volume, we investigated whether AS affects the proliferation or apoptosis of BC cells. The cells were treated by AS at different doses reported in a previous study [22]. Real-time PCR revealed that AS at a concentration of 20 μM, but not at 5 μM and 10 μM, significantly downregulated the mRNA levels of ki-67 and Bcl-2 in MDA-MB-231 cells (Figure 2A). Flow cytometry indicated that in the AS-treated group, BrdU incorporation in MDA-MB-231 cells was markedly decreased, and the percentage of apoptotic cells was significantly increased (Figure 2B,C). Similarly, AS inhibited the proliferation of 4T1 cells and promoted their apoptosis (Figure 2D–F). To investigate whether AS inhibited the growth of BC cells in vivo, BC cells were labeled and injected into the tibias. The BrdU assay indicated that AS significantly decreased the proliferation of MDA-MB-231 cells and 4T1 cells in vivo (Figure 2G,H). These results suggest that AS can inhibit proliferation and induce the apoptosis of BC cells both in vivo and in vitro.

### 3.3. AS Inhibited the Growth of BC Cells by Upregulating Ddit3

**Figure 3:**
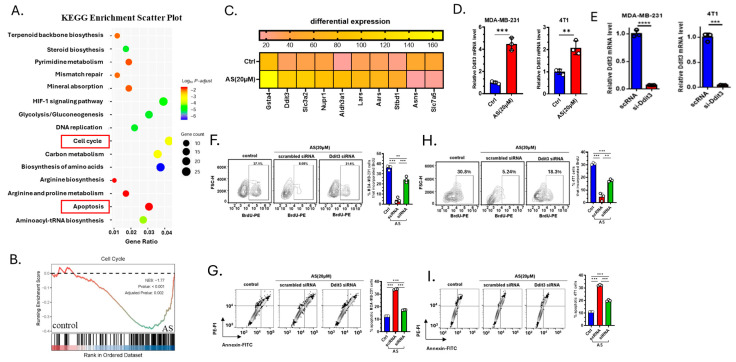
Ddit3 is a key regulator of AS-mediated inhibition of BC cell growth. (**A**) KEGG enrichment analysis showing differentially expressed pathways in the AS-treated group compared to the control group. (**B**) GSEA indicating that the cell cycle pathway was significantly inhibited in the AS-treated group compared to the control group. (**C**) Heatmap of the top 10 differentially expressed genes. (**D**) Real-time PCR analysis detecting the mRNA levels of Ddit3 in MDA-MB-231 (**left**) or 4T1 cells (**right**) after treatment with AS or vehicle. (**E**) The mRNA level of Ddit3 in MDA-MB-231 cells and 4T1 cells transfected with si-Ddit3 was detected by real-time PCR. (**F**,**H**) Representative flow cytometry images of BrdU incorporation in MDA-MB-231 (**F**) and 4T1 (**H**) cells after treatment with AS with or without transfection of Ddit3 siRNA (**left**) and quantification (**right**). (**G**,**I**) Representative flow cytometry images of apoptotic MDA-MB-231 (**G**) and 4T1 (**I**) cells after treatment with AS with or without Ddit3 knockdown (**left**) and the quantification results (**right**). AS, astragalin; ctrl, control. ** *p* < 0.01; *** *p* < 0.001. **** *p* < 0.0001.

To investigate how AS inhibits the growth of BC cells, 4T1 cells treated with AS or the vehicle were analyzed through RNA-sequencing. Kyoto Encyclopedia of Genes and Genomes (KEGG) analysis identified genes enriched in the cell cycle pathway and apoptosis pathway (Figure 3A). Gene Set Enrichment Analysis (GSEA) also showed that the cell cycle pathway was significantly downregulated in the AS-treated group (Figure 3B). Among differentially expressed genes, the mRNA expression level of Ddit3 was significantly upregulated in the AS-treated group, which was reported to be associated with cellular apoptosis and proliferation (Figure 3C). Real-time PCR analysis confirmed that the mRNA level of Ddit3 in MDA-MB-231 cells and 4T1 cells was significantly upregulated in the AS-treated group (Figure 3D). Then, the knockdown of Ddit3 was demonstrated at the mRNA level in MDA-MB-231 cells and 4T1 cells (Figure 3E). Consistently, Ddit3 knockdown reversed the effects of AS on the proliferation (Figure 3F,H) and apoptosis (Figure 3G,I) of BC cells. These results suggest that Ddit3 is a key downstream mediator of the effects of AS on the growth of BC cells.

### 3.4. The IRE1-AKT Pathway Regulates the Expression of Ddit3 in BC Cells

**Figure 4:**
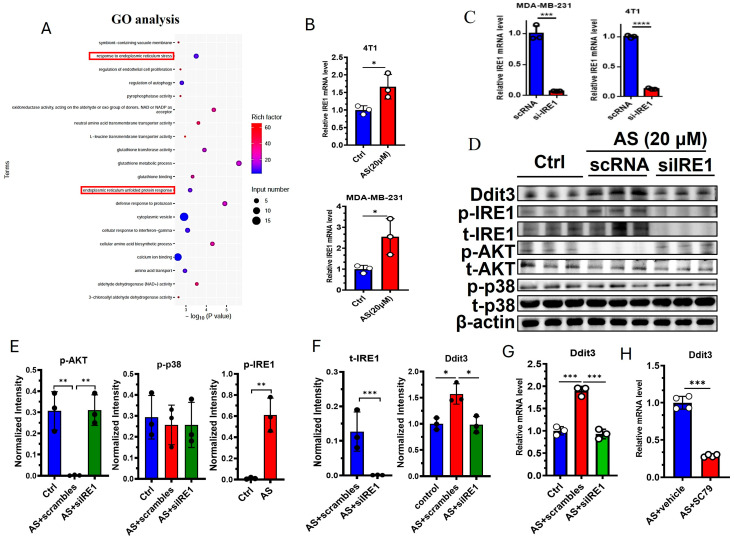
AS inhibited the expression of Ddit3 by regulating the IRE1-AKT pathway. (**A**) GO analysis indicating that ER stress pathways significantly changed in the AS-treated group. (**B**) Real-time PCR analysis detecting the mRNA levels of IRE1 in MDA-MB-231 cells or 4T1 cells. (**C**) The mRNA level of IRE1 in MDA-MB-231 cells and 4T1 cells transfected with si-IRE1 was detected by real-time PCR. (**D**) Western blotting detecting the levels of total and phosphorylated IRE1, AKT, and p38 after treatment with AS with or without IRE1 knockdown. (**E**,**F**) Quantification of C. (**G**) Real-time PCR analysis detecting the mRNA levels of Ddit3 in 4T1 cells treated with AS with or without IRE1 knockdown. (**H**) Real-time PCR analysis detecting the mRNA levels of Ddit3 in 4T1 cells treated with AS in the absence or presence of SC79. AS, astragalin. * *p* < 0.05; ** *p* < 0.01, *** *p* < 0.001.

GO analysis indicated that “response to endoplasmic reticulum stress” and “endoplasmic reticulum unfolded protein response” were significantly changed in the AS-treated group compared to the control group (Figure 4A). Interestingly, Ddit3 was found to be involved in endoplasmic reticulum stress; thus, we hypothesized that endoplasmic reticulum stress may regulate the expression of Ddit3 after treatment with AS. As expected, the mRNA expression of IRE1 was significantly upregulated in 4T1 cells and MDA-MB-231 cells (Figure 4B). Then, the knockdown of IRE1 was demonstrated at the mRNA level in MDA-MB-231 cells and 4T1 cells (Figure 4C). Western blotting indicated that the protein levels of Ddit3 and the phosphorylation levels of IRE1 were markedly upregulated in the AS-treated group (Figure 4D,E, Supplementary Document S1–S4). Since the ATK pathway and the p38 MAPK pathway are downstream of IRE1, we detected the activation status of these pathways after IRE1 knockdown. IRE1 knockdown significantly downregulated the protein levels of total IRE1 (Figure 4D,E). In addition, treatment with AS significantly inhibited the levels of phosphorylated AKT but had little effect on p38 MAPK activation (Figure 4D,F). In addition, phosphorylated AKT was upregulated after IRE1 knockdown (Figure 4D,F). These results suggest that AS can regulate the activation of IRE1 and the AKT pathway in 4T1 cells. Furthermore, IRE1 knockdown was found to reverse the effects of AS on the mRNA expression of Ddit3 (Figure 4G). Similarly, activation of the AKT pathway reversed the effects of AS on the mRNA expression of Ddit3 (Figure 4H). Taken together, we found that the IRE1-AKT pathway plays a key role in AS-mediated regulation of Ddit3 expression in 4T1 cells.

### 3.5. AS Inhibited Osteoclastogenesis in the Bone Metastasis of BC

**Figure 5:**
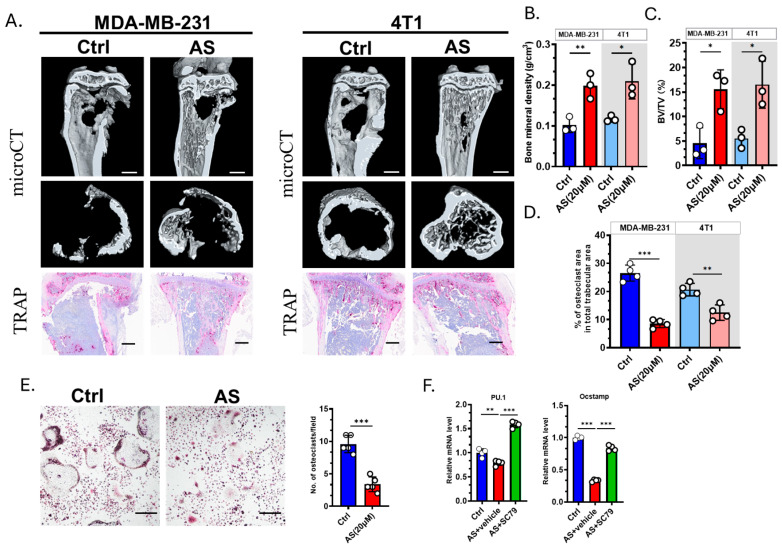
AS inhibited osteoclastogenesis and attenuated bone destruction. (**A**) Representative images of microCT (**upper**; scale bar = 1 mm) or TRAP staining (**lower**; scale bar = 100 μm) detecting bone destruction after injecting MDA-MB-231 cells or 4T1 cells in the AS-treated group or control group. (**B**) Quantification of bone mineral density in microCT analysis in A. (**C**) Quantification of bone volume/tissue volume (BV/TV) in microCT in A. (**D**) Quantification of the number of osteoclasts after TRAP staining in A. (**E**) TRAP staining for detecting the number of osteoclasts in the AS-treated group and the control group (**left**, Scale bar = 200 μm) and the quantification results (**right**). (**F**) Real-time PCR analysis showing the mRNA levels of osteoclastogenic biomarkers in the AS-treated group in the absence or presence of SC79. AS, astragalin; ctrl, control. * *p* < 0.05; ** *p* < 0.01; *** *p* < 0.001.

We next explored whether AS affected the severity of osteolysis. microCT revealed that AS can effectively prevent bone destruction (Figure 5A–C). TRAP staining showed that the number of OCs was significantly decreased in the AS-treated group compared to the control group (Figure 5A,D). Furthermore, we confirmed that AS negatively regulates OC formation in vitro (Figure 5E). The expression of osteoclastogenesis biomarkers was significantly downregulated in the AS-treated group compared to the control group. This effect was reversed by SC79, an AKT agonist (Figure 5F). Our findings indicated that AS can directly inhibit OC formation both in vitro and in vivo.

### 3.6. AS Inhibited Osteoclastogenesis Partly by Inhibiting the AKT Pathway

**Figure 6:**
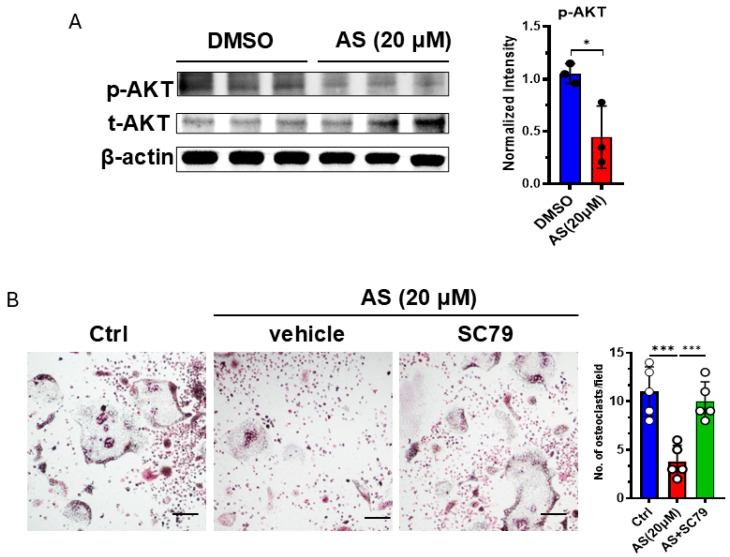
AS inhibited osteoclast formation by regulating the AKT pathway. (**A**) Western blotting for detecting the levels of phosphorylated and total AKT in the AS-treated group or the control group (**left**) and the quantification results (**right**). (**B**) TRAP staining for detecting the number of osteoclasts after stimulation with AS in the absence or presence of SC79 (**left**; scale bar = 200 μm) and the quantification results (**right**). AS, astragalin. * *p* < 0.05; *** *p* < 0.001.

Since we found that AS can regulate the AKT pathway, and this pathway was reported to be involved in osteoclastogenesis, we detected the activation of the AKT pathway in OCPs after treatment with AS or vehicle. As expected, treatment with AS significantly inhibited AKT phosphorylation in OCPs (Figure 6A). Furthermore, the AKT agonist, SC79, reversed the effects of AS on osteoclastogenesis (Figure 6B). These results suggest that AS inhibited OC formation partly by inhibiting AKT activation.

## 4. Discussion

BC and its complications have been challenging worldwide. Recent studies have increasingly attempted to promote the therapeutic outcomes by adopting new strategies, such as minimally invasive techniques [23,24]. In addition, natural components of plants were found to be promising agents for adjuvant therapy. AS is an effective flavonoid with therapeutic effects across multiple diseases [25]. Generally, it possesses anti-inflammatory properties and promotes tissue regeneration [25,26,27]. Furthermore, AS was found to prevent inflammatory osteolysis [28,29], suggesting that AS can affect bone homeostasis. In this study, we found that AS can attenuate tumor-associated osteolysis, at least in the bone metastasis of BC. Compared to inflammatory osteolysis, tumor-associated osteolysis showed a more complex mechanism. In addition to inflammatory cytokines, other bioactive ingredients, such as tumor cell metabolites and chemokines, are involved in the formation of the tumor microenvironment. Thus, our findings suggest that AS can prevent bone loss. As previously mentioned, although current therapies for treating bone metastasis of BC focus on inhibiting the function of osteoclasts and bone loss, tumor cells are hardly cleared directly. Here, we found that AS acted as a potent bioactive ingredient inducing cell death in both human and murine BC cells, showing promising capacity to be utilized as an adjuvant treatment of current therapies used in clinics to help restrict the viability of tumor cells, directly inhibit the growth of tumor, and prevent bone destruction.

Transcriptomic analysis revealed that Ddit3 is a key regulator of AS-mediated BC cell death. Ddit3 can induce the apoptosis of various cell types, including BC cells, which is consistent with our findings [30,31,32]. Notably, GO analysis revealed that endoplasmic reticulum (ER) stress was significantly changed after treatment with AS. Furthermore, we found that knocking down IRE1, the key regulator of ER stress, can significantly inhibit the expression of Ddit3, suggesting that AS may regulate BC cell death through Ddit3 and ER stress. Consistently, it was previously reported that Ddit3 is involved in ER stress-induced apoptosis of lung cancer cells [30]. The AKT and p38 MAPK pathways were reported to be associated with ER stress and Ddit3 expression; however, we found that IRE1 significantly regulated AKT, but not p38 MAPK, after treatment with AS. Some studies reported that the p38 MAPK pathway could regulate the expression of Ddit3 in BC cells [33,34]. Together, these findings suggest that Ddit3 is regulated by distinct pathways based on the circumstance, and AS promotes the expression of Ddit3, mainly through the AKT pathway.

## 5. Conclusions

Targeting osteoclastogenesis is a key strategy for improving the outcome of bone metastasis in BC; however, there is a lack of effective therapies to limit the viability of tumor cells. Our results indicated that AS could exert dual effects on both the tumor microenvironment and the growth of tumor cells. We firstly found that the cell death of BC cells could be induced by AS in a Ddit3-based manner. The AKT pathway was important in mediating the effects of AS on both the cell death of BC cells and osteoclastogenesis. In summary, we found that AS can promote the apoptosis of BC cells through ER stress-mediated apoptosis and inhibit osteoclastogenesis. AS is a promising treatment for the bone metastasis of BC. Here, we found that AS can simultaneously target cancer cells and osteoclasts, making it a promising adjuvant therapy for bone metastasis in BC.

## Data Availability

The data supporting this report’s findings are available from the corresponding author upon reasonable request. The RNA-Seq data have been submitted to the NCBI Gene Expression Omnibus (GEO) (https://www.ncbi.nlm.nih.gov/geo/query/acc.cgi?acc=GSE273845) (accessed on 31 August 2025) under accession numbers GEO: GSE273845.

## Supplementary Materials

The following supporting information can be downloaded at: https://www.mdpi.com/article/10.3390/cancers17213442/s1, Supplementary Document S1–S4.
