# Supplementary figures and images for: Astragalin Attenuates Bone Destruction and the Progression of Bone Metastasis in Breast Cancer
Source: Cancers (Basel). 2025 Oct 27;17(21):3442. doi: 10.3390/cancers17213442 (PMC12606754; doi:10.3390/cancers17213442)

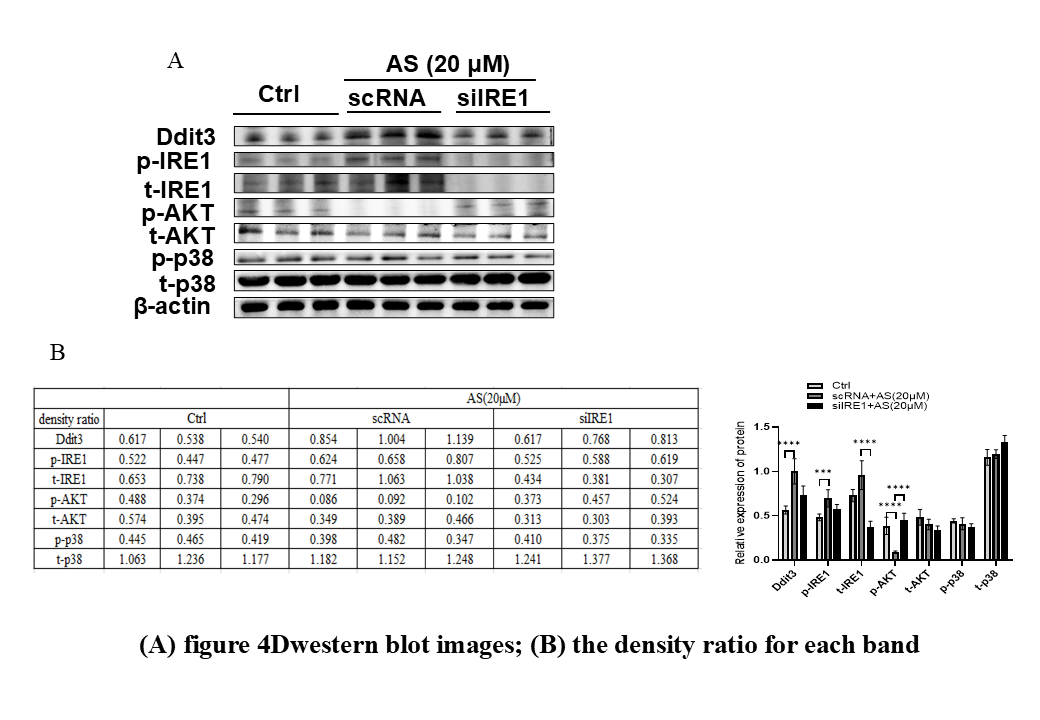

Supplement: Supplementary file 1 [file cancers-17-03442-s001.zip › Supplementary Document S1.TIF]

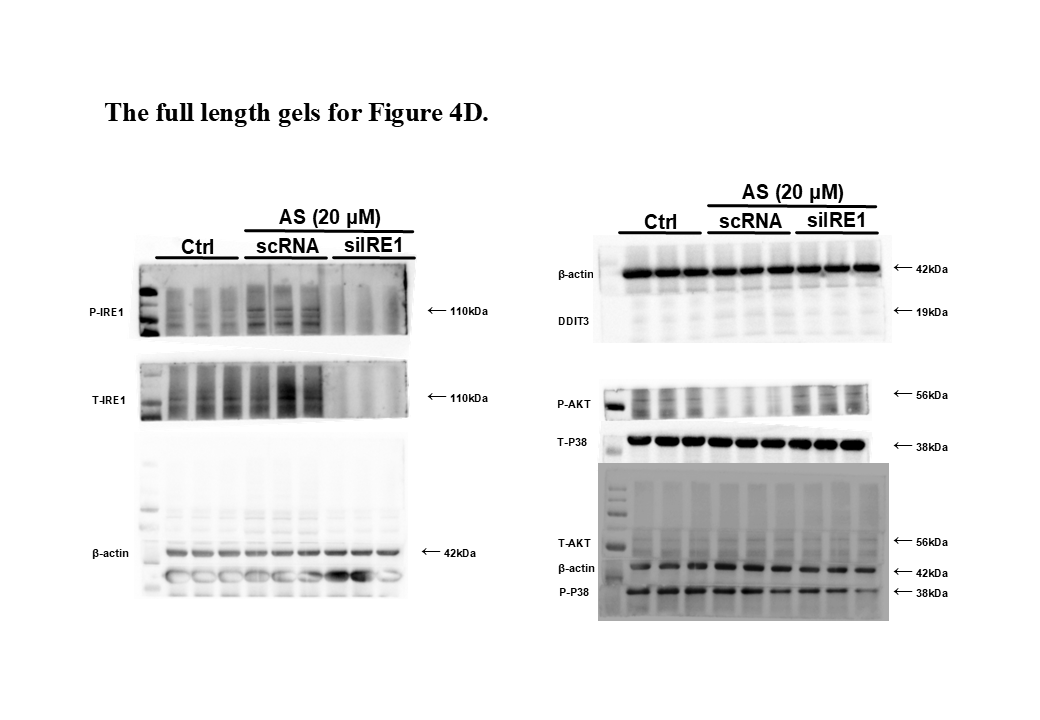

Supplement: Supplementary file 1 [file cancers-17-03442-s001.zip › Supplementary Document S2.TIF]

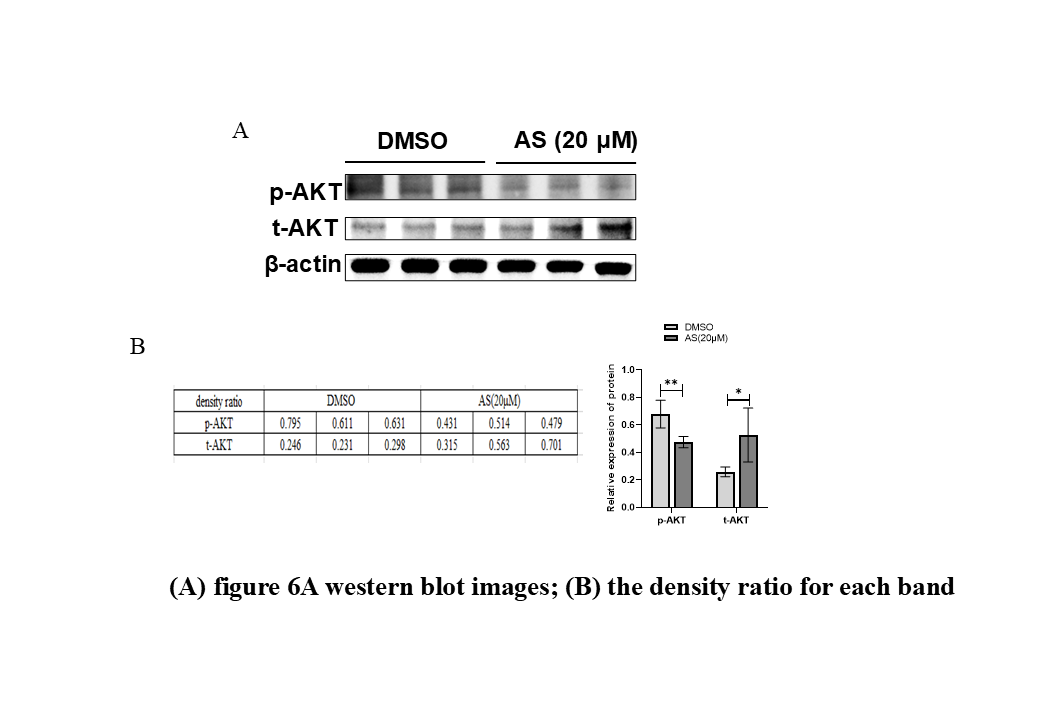

Supplement: Supplementary file 1 [file cancers-17-03442-s001.zip › Supplementary Document S3.TIF]

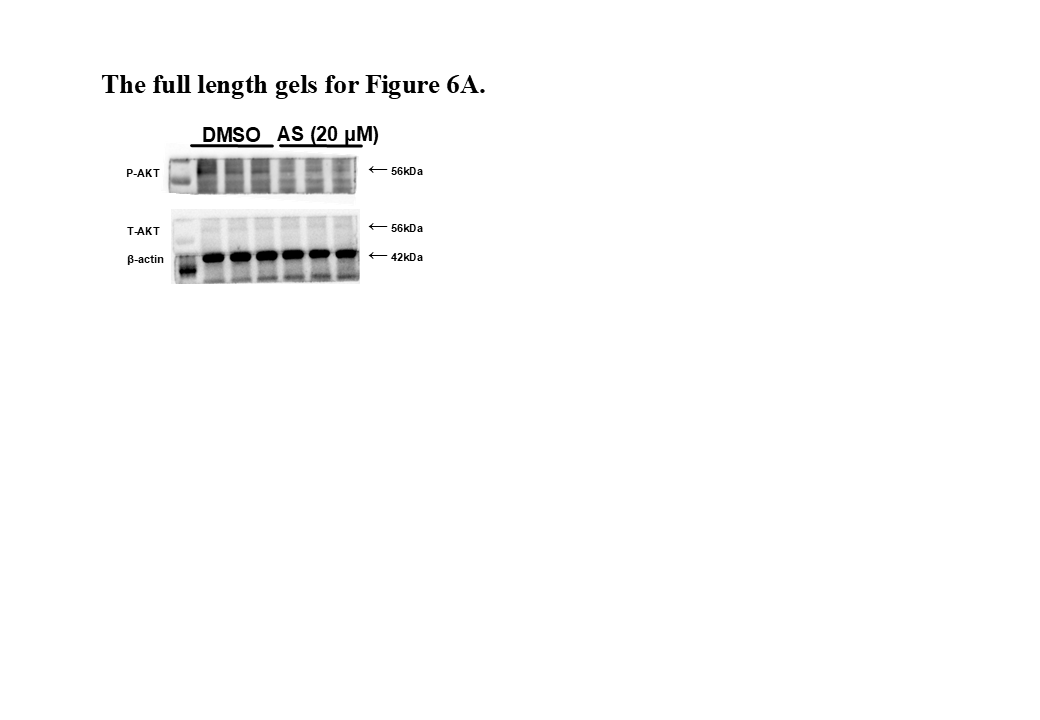

Supplement: Supplementary file 1 [file cancers-17-03442-s001.zip › Supplementary Document S4.TIF]
